# The Ideal Method of Administering Colchicum and Applying Methyl Salicylate Locally in Rheumatic Conditions

**Published:** 1907-01

**Authors:** M. R. Dinkelspiel

**Affiliations:** Philadelphia, Pa.


### The Ideal Method of Administering Colchicum and Applying Methyl Salicylate Locally in Rheumatic Conditions.

BY M. R. DINKELSPIEL, M. D., PHILADELPHIA, PA.

The accurate determination of the exciting factor of rheumatic conditions, and, in fact, most of the errors of metabolism, has not yet been reached. We know of many predisposing factors, such as heredity, deficient elimination, sedentary life, consumption of an excess of nitrogenous food, especially when associated with alcohol, etc., etc. We have surmised, and perhaps correctly so, that acute rheumatic fever is due to bacterial invasion. Yet it can not be denied that we have made by far more progress in the therapy of these conditions than in the determination of their etiology.

Chemical and physiological experiments, pathological examinations, and the most careful clinical observations—all have failed to accord rheumatic and gouty processes a definite and accurate position, as far as their exciting cause and correct etiology are concerned. These disorders occupy somewhat an analogous position to the subject of syphilis, in which we possess a specific, while the etiological factor remains obscured. Based upon our knowledge of the signs of defective metabolism that accompany the various stages of rheumatic processes, we advise our patients to modify their diet, stimulate their emunctories by attention to exercise, baths, the drinking of quantities of water and giving proper attention to the bowels.

In my opinion, however, the nearest approach to therapeutic accuracy in some of these conditions that we have reached is in the employment of colchicine internally and the external application of the oil of wintergreen. Both of these drugs I have found must be used absolutely pure or they are worse than useless. For some time I have used, and propose to continue to use, the colchicine in the form of colchisal capsules, which investigation has shown to contain the equivalent of three minims of pure methyl salicylate and one two-hundred-and-fiftieth of a grain of pure colchicine and the same amount of a specially pure and active extract of cannabis indica, which is not obtainable in commerce, however. These capsules I have found to be prepared on a most thoroughly scientific basis, and are absolutely reliable. For local swellings, as in acute arthritis of rheumatic origin, in gouty attacks, in myalgias of all description, especially lumbago, torticollis and pleurodynia, as well as in sciatica, I have invariably employed the mentho-methyl-oleo-salicyalte, known as betul-ol. Its peculiarly efficient power of penetration and the purity of its ingredients have stood me in good stead. The following cases plainly indicate the peculiar specific value of these two products. They are not new drugs, but scientifically accurate dispensations of well-known ingredients, the purity and efficacy of which vary at almost every drug store.

CASE 1.—Mrs. K., a German woman, aged 44 years, was of good health with the exception of rather frequent attacks of acute arthritis of rheumatic origin, She had taken salicylic acid in various combinations, and had frequently used linaments containing oil of wintergreen—at least, the prescriptions called for ol. gaultheriæ. I modified her diet, prescribed exercise, baths and laxatives, and ordered her to take one capsule of colchisal every hour for one day, one every two hours on the second day and one every four hours for a period of a week. Locally I ordered betul-ol applied by means of gentle friction for five minutes by the watch, twice daily. In ten days all symptoms disappeared, and for the past five months have not reappeared. In addition to the disappearance of the pain and swelling, she has gained in weight and has regained much energy and spirit, factors which so often go hand in hand with improvement in lithemic conditions. I have advised her to take one capsule three times daily for one week during each month, to prevent any recurrence.

CASE 2.—Mrs. R., aged 30 years, widow, consulted me for very acute pain in the left side. She had had pleurisy two years before. Examination showed the pain to be due to muscular origin; in short, a typical case of myalgia. I prescribed colchisal capsules to point of tolerance, and ordered betul-ol to be gently rubbed over the painful area twice daily. The pain disappeared in three days. It had been my practice to strap the chest in these conditions, with great benefit. But a patient otherwise in good health, as a rule, does not like the strips around the chest. I have found the above described porcedure a valuable substitute, with the additional value of preventing further attacks.

CASE 3.—Miss A. L., aged 19 years, was attacked severely with acute rheumatic fever. Both knees were greatly swollen, and her general condition was very poor. She had a number of successive drenching sweats. Her temperature fluctuated between 101° to 104° F., and at one time threatened to reach a point of hyperpyrexia.

It had been my invariable custom up to the time of this case to administer salicylate of sodium internally and apply equal parts of wintergreen and olive oil locally to the inflamed joints. However, in this case, encouraged by previous observations and experiences, I ordered colchisal capsules, one every hour for twenty-four hours, and one every three hours thereafter. Locally I applied betul-ol and olive oil, equal parts, and covered the inflamed joints with lambs' wool over oiled silk. With each capsule I ordered a glass of cool Vichy to be given, and confined the patient to a milk diet. The case made a rapid, uneventful and uncomplicated recovery without sequelæ.

